# 

**Published:** 1908-04

**Authors:** 


### EDITORIAL DEPARTMENT.

### ☞ VERY IMPORTANT NOTICE. ☜

*To My Subscribers:*

A recent ruling of the Postoffice Department *compels* publishers to drop all subscribers who (in case of monthly publications) are even so much as four months in arrears for subscription. They must renew at once, or will not be recognized by the Department as subscribers, and copies addressed to such after April will be refused the mails unless a stamp (about 3 cents each for the "Red Back") is affixed to each. This is out of the question. The law will be enforced. This will entail heavy loss on publishers unless subscribers act promptly. I believe my "old guard" appreciate the "Red Back" and wish it continued, and I hope and expect that they will rally to its support. *Don't delay.* It is vital. I have been very indulgent and have pushed none for payment, feeling assured that they would remit at convenience, as they have done in the past for many years. I am up against it now, good and hard, and it is simply a case of *must.* I have no discretion,—*it is the law.* Bills have been sent (or will be soon) to all who are in arrears, and I hope for a prompt response. Your personal check will do.

The law reads: "Expired subscriptions: A reasonable time will be allowed publishers (monthly journals, four months) to secure renewal of subscriptions, but unless subscriptions are expressly renewed after the term for which they are paid, they shall not be counted in the legitimate list of subscribers, and copies mailed on account thereof shall not be accepted for mailing at the second-class postage rate," etc. (It goes into effect April 1, 1908.)

